# Relationship between insulin-like growth factor-1 and cerebral small vessel disease and its mechanisms: advances in the field

**DOI:** 10.3389/fnagi.2023.1190869

**Published:** 2023-06-08

**Authors:** Hao Du, Jian Xia, Lei Huang, Lan Zheng, Wenping Gu, Fang Yi

**Affiliations:** ^1^Department of Neurology, Xiangya Hospital, Central South University, Changsha, Hunan, China; ^2^Clinical Research Center for Cerebrovascular Disease of Hunan Province, Central South University, Changsha, Hunan, China; ^3^National Clinical Research Center for Geriatric Disorders, Xiangya Hospital, Central South University, Changsha, Hunan, China; ^4^Department of Rehabilitation, Hunan Provincial People’s Hospital, The First Affiliated Hospital of Hunan Normal University, Changsha, Hunan, China; ^5^Department of Geriatric Neurology, Xiangya Hospital, Central South University, Changsha, Hunan, China

**Keywords:** insulin-like growth factor-1, cerebral small vessel disease, neurovascular unit, blood–brain barrier, microvascular rarefaction

## Abstract

Insulin-like growth factor-1 (IGF-1) is an active polypeptide protein that closely resembles the structural sequence of insulin and is involved in a variety of metabolic processes in the body. Decreased IGF-1 circulation levels are associated with an increased risk of stroke and a poorer prognosis, but the relationship with cerebral small vessel disease (cSVD) is unclear. Some studies found that the level of IGF-1 in patients with cSVD was significantly reduced, but the clinical significance and underlying mechanisms are unknown. This article reviews the correlation between IGF-1 and cerebrovascular disease and explores the potential relationship and mechanism between IGF-1 and cSVD.

## 1. Introduction

Cerebral small vessel disease (cSVD) is a common neurological disorder in elderly individuals that can lead to acute and chronic clinical syndromes, such as stroke and cognitive impairment, seriously affecting quality of life (Litak et al., 2020). Moreover, it has been shown that abnormal glucose metabolism may be an important risk factor for cSVD. Specifically, cSVD, particularly cerebral microhemorrhage, is more common in young people with type 1 diabetes than in healthy controls (Thorn et al., 2019). Furthermore, carotid-femoral pulse wave velocity and reactive hyperemia index values were higher and lower, respectively, in diabetic foot patients than in control diabetic patients without diabetic foot and healthy controls (Tuttolomondo et al., 2017), indicating endothelial dysfunction in diabetes.

Insulin-like growth factor-1 (IGF-1) is a bioactive peptide with high structural sequence similarity to insulin, involved in various metabolic processes in the body; furthermore, it is an important nutritional, growth, angiogenic and anti-apoptotic factor with neuroprotective effects on neuroinflammation and excitotoxicity (Wrigley et al., 2017; Hayes et al., 2021b). IGF-1 participates in various anti-inflammatory and pro-inflammatory processes *in vivo*, such as affecting the direction of polarization of microglia (Labandeira-Garcia et al., 2017). Moreover, neuroinflammation is an important link of stroke pathogenesis and affects the prognosis (Tuttolomondo et al., 2016). Studies have shown a negative correlation between age and circulating IGF-1 levels in humans and rodents, associated with a high risk of stroke and poor outcomes (Johnsen et al., 2005; Saber et al., 2017; Zhang et al., 2019; Lee et al., 2021). Additionally, altered IGF-1 levels are associated with an increased risk of multiple age-related neuropathies characterized by neurovascular dysfunction, excitotoxicity, and oxidative stress (Sonntag et al., 2013; Gubbi et al., 2018; Hayes et al., 2021a). However, the characteristics of clinically relevant changes in IGF-1 in cSVD remain unclear. Some studies have reported decreased IGF-1 levels in patients with cSVD and an association with cognitive impairment (Kang et al., 2021); however, the exact mechanism is not known. This article reviewed the potential relationship between IGF-1 and cSVD and its mechanisms.

## 2. Relationship between IGF-1 and cerebral vessel disease

### 2.1. IGF-1 and ischemic stroke

IGF-1 is associated with atherosclerosis and vascular disease development. For example, Framingham et al. found that circulating IGF-1 levels negatively correlate with ischemic stroke incidence, especially in patients with insulin resistance (Saber et al., 2017). Several clinical studies have found that patients with low circulating IGF-1 levels at admission have a higher mortality rate and are associated with increased stroke scale scores from the National Institute of Health several months after stroke (Bondanelli et al., 2006; De Smedt et al., 2011). A longitudinal study of chronic ischemic stroke survivors found that high IGF-1 levels 3 months post-stroke were associated with better Modified Rankin Scale scores (Åberg et al., 2011). Moreover, human IGF-1 levels are negatively correlated with the development of post-stroke depression (Zhang et al., 2018; Table 1).

**Table 1:** The main findings of clinical studies on insulin-like growth factor-1 and cerebral vessel disease.

Cerebral vessel disease type	Patient characteristics	Main findings	References
Incident ischemic stroke	254 patients and 254 matched controls; a nested case–control study	Participants in the bottom quartiles of insulin-like growth factor-1 (IGF-I) and insulin-like growth factor-binding protein 3 (IGFBP-3) levels (median concentrations, 72 and 2,937 ng/mL, respectively) are at increased risk of ischemic stroke. The IGF axis may be involved in the pathogenesis of ischemic stroke.	Johnsen et al. (2005)
Incident ischemic stroke	757 patients without prevalent stroke (mean age 79 ± 5, 62% women) prospectively followed up for an average of 10.2 years	Higher IGF-1 levels are associated with a lower risk of incident ischemic stroke.	Saber et al. (2017)
Acute hemispheric ischemic stroke	255 patients (age ≥ 18) who presented with substantial neurological deficit within 6 h after acute hemispheric ischemic stroke onset	Higher serum IGF-1 levels just after ischemic stroke onset are associated with a better neurological and functional outcome.	De Smedt et al. (2011)
Acute stroke	85 patients (mean age 83 ± 7.4, 34% male) and 88 matched controls	Mean IGF-1 levels are lower in patients with stroke than in controls (69 ± 45 ng/mL vs. 102 ± 67 ng/mL, P adjusted for age = 0.001). IGF-1 levels are negatively correlated with poor outcome.	Denti et al. (2004)
Ischemic stroke	407 patients (mean age 55, 260 males, 147 females), and 40 matched controls	Higher serum IGF-1 levels during the rehabilitation phase of stroke correlate to better recovery of long-term function.	Åberg et al. (2011)
First-ever acute ischemic stroke	225 patients (mean age 67 ± 5, 49.8% male) and 120 matched controls prospectively followed up for 1 year	Lower serum IGF-1 levels at admission are associated with a higher risk of developing post-stroke depression.	Zhang et al. (2018)
Early retinopathy	268 patients (mean age 13.03 ± 1.9, 116 males) with overweight/obesity; a cross-sectional study	Relatively high levels of IGF-1 during childhood and adolescence may act as an additional risk factor for microvascular damage.	Bizzarri et al. (2019)
Diabetic retinopathy (DR)	46 patients(mean age 14.3 ± 4.36, 40% male) with type 1 diabetes; a cross-sectional study for 2 years	Severity of DR in patients with type 1 diabetes is negatively correlated with serum IGF-1 levels.	Raman et al. (2019)
Diabetic retinopathy	806 patients (aged 1 to 18) with a type 1 diabetes duration of at least 6 months; a longitudinal study for 6 years	A low individual mean IGF-I level is associated with progression of retinopathy.	Öberg et al. (2018)
Cerebral small vessel disease (CSVD)	216 patients (mean age 67.57 ± 8.53 years, 31.9% female)	Lower serum IGF-1 levels are associated with a higher risk of cognition impairment in a patient with CSVD.	Kang et al. (2021)

### 2.2. IGF-1 and cSVD

#### 2.2.1. Clinical correlation between IGF-1 and cSVD

As an extension of the central nervous system (CNS), the retina exhibits a feature similar to the brain in terms of anatomy and physiological function, including microvascular structures (London et al., 2013). Studies have shown that patients with cSVD often present retinal changes associated with cerebral changes, such as the foveal outer plexiform layer volume being positively correlated with the number and volume ratio index of white matter lesions, and the temporal peripapillary retinal nerve fiber layer thickness being negatively correlated with it (Langner et al., 2022). IGF-1 is involved in the pathological process of retinal degeneration and may be associated with the microglia-mediated inflammatory response and insulin resistance (Arroba et al., 2018). A single-center cross-sectional study of overweight children and adolescents reported that IGF-1 levels were significantly elevated in patients with early atherosclerotic retinopathy independent of age, sex, and family history of type 2 diabetes, obesity, cardiovascular disease, hypertension, and dyslipidemia (Bizzarri et al., 2019). In contrast, in a study of adolescent patients with type 1 diabetes, retinopathy severity was negatively correlated with serum IGF-1 levels (Öberg et al., 2018; Raman et al., 2019; Table 1). However, not every vascular pathological sign seen in the cerebral due to IGF-1 deficiency will be shown in the retina. Induction of chronic hypertension in an IGF-1-deficient mouse model exacerbated the development of cerebral microhemorrhage, with retinal vascular abnormalities and gliosis, but without signs of retinal hemorrhage and retinal degeneration (Miller et al., 2022). Thus, IGF-1 might not be a good biomarker for using retinal changes as cerebrovascular changes.

cSVD is an important cause of vascular cognitive impairment (VCI). In humans, IGF-1 levels are positively correlated with hippocampal volume and memory (Maass et al., 2016; Norling et al., 2020). A study of patients with cSVD showed a significant correlation between significantly low IGF-I levels and Montreal Cognitive Assessment scores in patients with cognitive impairment (Kang et al., 2021). Another study of patients with cognitive impairment showed that patients with low IGF-1 levels showed dramatically lowered Mini-Mental State Examination scores over time (Vidal et al., 2016). Additionally, IGF-1 gene therapy was found to improve cognitive function in a mouse model of ischemic stroke (Zhu et al., 2008; Hayes et al., 2021b), suggesting that IGF-1 may play a role in cognitive impairment due to cSVD.

#### 2.2.2. Effect of IGF-1 on cSVD-related imaging

A few studies have investigated the effect of IGF-1 on cSVD-related imaging indicators. A study found that patients with brain white matter injury with high baseline serum IGF-1 had better recovery (Feeney et al., 2017). A fetal sheep ischemic model showed that exogenous IGF-1 may decrease the loss and demyelination of postischemic oligodendrocytes and increase gliosis by inhibiting postischemic programmed cell death, thus playing a role in white matter protection (Guan et al., 2001). There are many similarities between cerebral microbleeds (CMBs) observed in IGF-1-deficient mice and in elderly hypertensive patients, including the relative size of the hemorrhage, clinical signs, and progressive nature of the pathological process. IGF-1 deficiency primarily increases the probability of cortical or subcortical CMBs (Toth et al., 2015b; Tarantini et al., 2017). In a study on the relationship between IGF-1, cognitive function, and neuroimaging indicators in elderly hypertensive patients, IGF-1 deficiency was found to be associated with hippocampal atrophy (Angelini et al., 2009). Another study showed that reduced serum IGF-1 levels were associated with brain atrophy and a high risk of Alzheimer’s disease in a healthy elderly population (Westwood et al., 2014).

## 3. Possible mechanism of IGF-1 in cSVD

### 3.1. Neurovascular units

**Figure 1:**
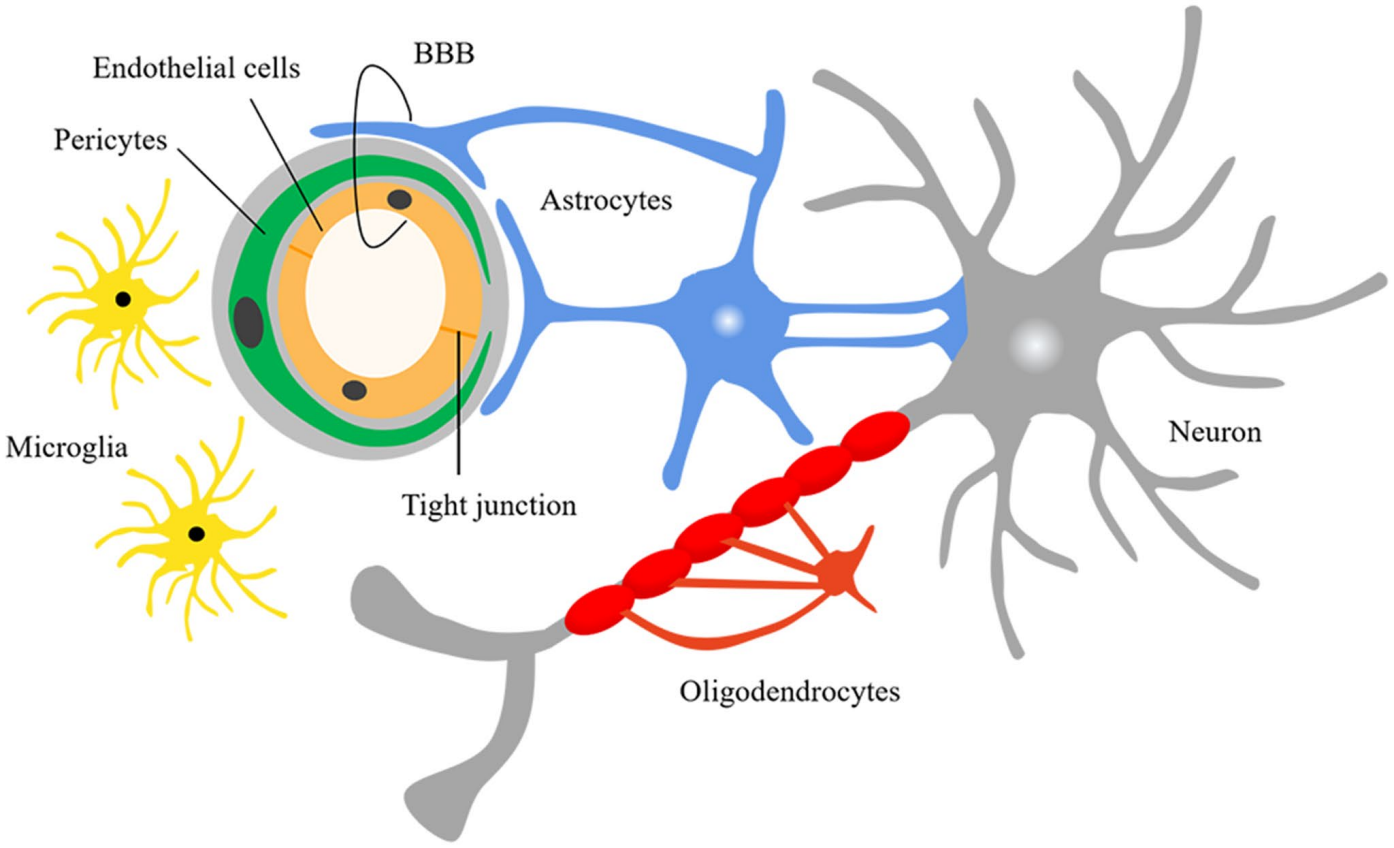
Neurovascular units. BBB, blood–brain barrier.

**Figure 2:**
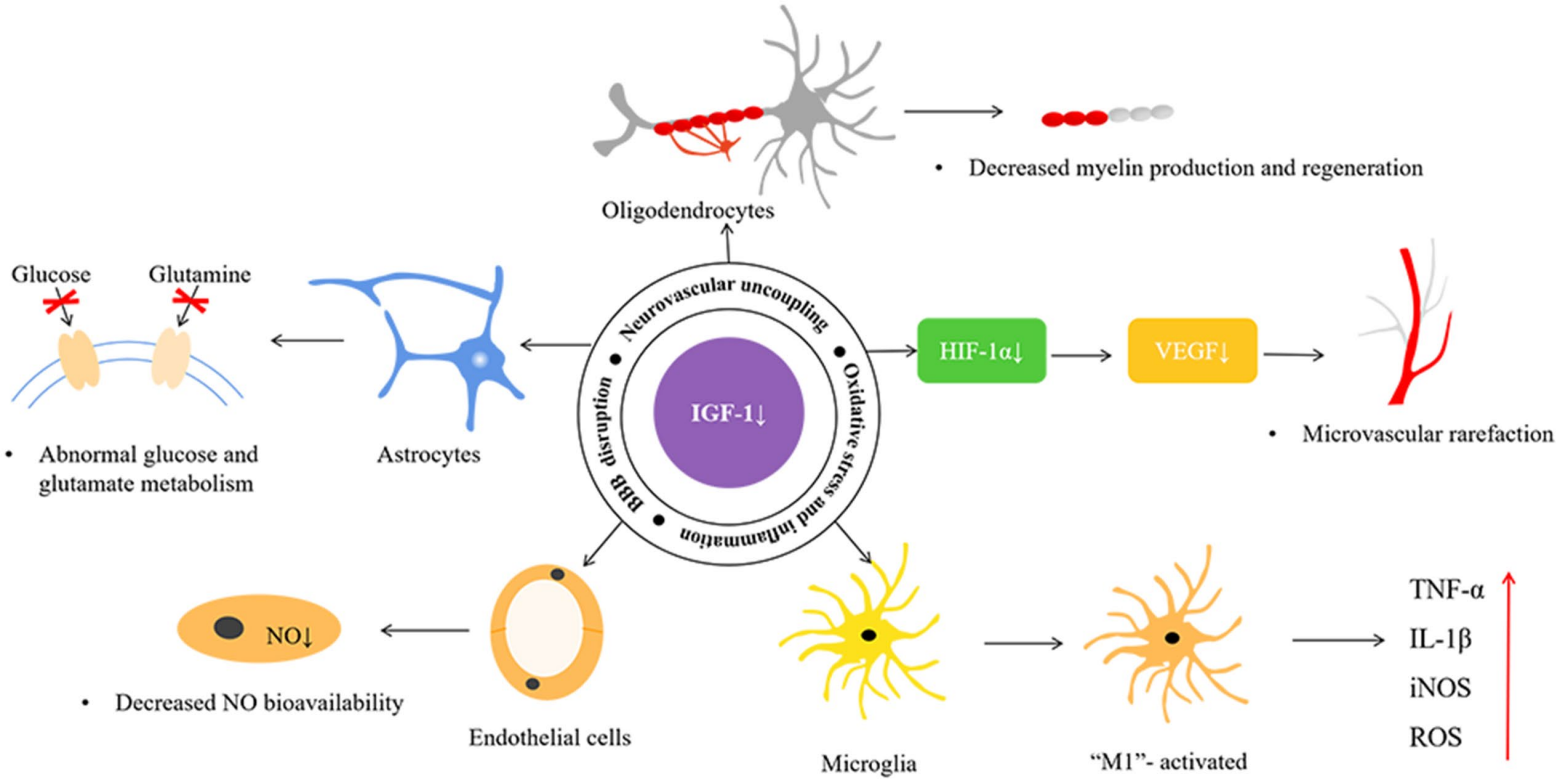
The possible mechanisms of insulin-like growth factor-1 and cerebral small vessel disease. HIF-1α, hypoxia-inducible factor-1α; VEGF, vascular endothelial growth factor; NO, nitric oxide; TNF-α, tumor necrosis factor-α; IL-1β, interleukin-1β; iNOS, inducible nitric oxide synthase; ROS, reactive oxygen species.

The NVU is the blood–brain barrier’s basic structural and functional unit and plays an important role in SVD pathogenesis, and it consists of neurons, astrocytes, vascular endothelial cells, pericytes, and vascular smooth muscle cells (Figure 1; Yang et al., 2022). Studies have shown that IGF-1 can affect various components of the NVU (Figure 2; Table 2).

**Table 2:** The main findings of possible mechanism of insulin-like growth factor-1 and cerebral small vessel disease.

Model	Experimental groups	Main findings	References
Igf1^f/f^; C57BL/6 male mice; Hypertension induced by ω-nitro-L-arginine-methyl ether (L-NAME) and angiotensin II (Ang II)	(1) Control + vehicle, (2) Control + Ang II, (3) Igf1^f/f^ + TBG-Cre-AAV8 + vehicle, (4) Igf1^f/f^ + TBG-Cre-AAV8 + Ang II	IGF-1 deficiency exacerbates hypertension-induced cerebral microbleeds (CMHs) in mice by Matrix metalloproteinases (MMP) activation, hypertrophy, and structural remodeling in cerebral vessels.	Tarantini et al. (2017)
Igf1^f/f^; C57BL/6 male mice	(1) Igf1^f/f^ + TBG-Cre-AAV8, (2) Control	IGF-1 deficiency impairs cerebromicrovascular endothelial function and mediation of cerebral blood flow (CBF) responses by glutamate and eicosanoid gliotransmitters.	Toth et al. (2015a)
Igf1^f/f^; C57BL/6 male mice; Hypertension induced by Ang II	(1) Control + vehicle, (2) Control + Ang II, (3) Igf1^f/f^ + TBG-Cre-AAV8 + vehicle, (4) Igf1^f/f^ + TBG-Cre-AAV8 + Ang II	IGF-1 deficiency reduces elasticity of cerebral arteries by reducing elastin content.	Fulop et al. (2019)
SD rats	(1) RAd-IGF-1, (2) RAd-DsRed	The IGF-1 gene therapy increases hippocampal astrocyte branching.	Pardo et al. (2016)
Romney-Suffolk fetal sheep; Bilateral brain injury induced by 30 min of reversible carotid artery occlusion.	(1) Sham, (2) Ischemia + vehicle, (3) Ischemia +3 μg rhIGF-1	Exogenous IGF-1 can reduce post-ischemic loss of oligodendrocytes and demyelination, probably by inhibiting post-ischemic programmed cell death.	Guan et al. (2001)
Romney-Suffolk fetal sheep; Bilateral brain injury induced by 30 min of reversible carotid artery occlusion.	(1) Sham, (2) Ischemia + vehicle, (3) Ischemia +3 μg rhIGF-1, (4) Ischemia +30 μg rhIGF-1, (5) Ischemia + (3 + 3) μg rhIGF-1	Exogenous IGF-1 can protect postischemic white matter, probably by suppressing late onset oligodendrocyte apoptosis, promoting oligodendrocyte regeneration, and increasing proliferation of reactive glia.	Cao et al. (2003)
Igf1^f/f^ and VE-Cadherin-Cre^ERT2^ mice	(1) Control, (2) Control + L-NAME, (3)VE-Cadherin-Cre^ERT2^/Igf1r^f/f^, (4) VE-Cadherin-Cre^ERT2^/Igf1r^f/f^ + L-NAME	Endothelium-specific disruption of IGF1R signaling impairs the endothelial NO-dependent component of Neurovascular coupling (NVC) responses.	Tarantini et al. (2021)
SD male rats; Carotid balloon injury by Clowes method	(1) Sham; (2) sham + IGF-1, (3) injury, (4) injury + IGF-1	Exogenous IGF-1 can protect vascular by inducing endothelial nitric oxide synthase (eNOS) expression in the neointima.	Cittadini et al. (2009)
CD-1 male mice; intracerebral hemorrhage (ICH) induced by collagenase or autologous blood injection	(1) Shem, (2) Vehicle, (3) 10 μg rhIGF-1, (4) 50 μg rhIGF-1, (5) AG1024 + 50 μg rhIGF-1, (6) Scramble siRNA +50 μg rhIGF-1, (7) IGF-1R siRNA +50 μg rhIGF-1	IGF-1 can decrease blood–brain barrier (BBB) permeability by reducing glycogen synthase kinase-3 (GSK3β) / mitogen-activated protein kinase kinase kinase 1 (MEKK1) activation and increasing the expression of tight junction proteins.	Nowrangi et al. (2019)
SD female rats; middle cerebral artery occlusion (MCAO)	(1) Ischemic + Control, (2) Ischemic + IGF-1, (3) Ischemic + IGF-1 + JB-1, (4) Non-ischemic + Control, (5) Non-ischemic + IGF-1, (6) Non-ischemic + IGF-1 + JB-1	Post-stroke IGF-1 treatment can decrease ischemic region and BBB permeability.	Bake et al. (2019)
Wistar Male rats	(1)Adult + Vehicle, (2) Adult + IGF-1, (3) Aged + Vehicle, (4) Aged + IGF-1	Post-stroke IGF-1 treatment can improve sensorimotor function in adults while it does not in aged rats, probably due to the different activation of microglial.	Serhan et al. (2019)
IGF-1R^+/−^; C57BL/6 mice; 1-methyl-4-phenyl-1,2,3,6-tetrahydropyridine (MPTP) challenge	(1) IGF-1R^+/−^ + MPTP, (2) IGF-1R^+/−^ + NaCl, (3) WT + MPTP, (4) WT + NaCl	IGF-1R^+/−^ mice after MPTP present more severe dopaminergic neuronal damage not caused by oxidative stress.	Nadjar et al. (2009)
Igf1^f/f^; C57BL/6 male mice; Hypertension induced by Ang II	(1) Control, (2) Control + Ang II, (3) Igf1^f/f^ + TBG-Cre-AAV8, (4) Igf1^f/f^ + TBG-Cre-AAV8 + Ang II	IGF-1 deficiency promotes microvascular rarefaction in the hippocampus and the neocortex and leads to structural maladaptation of the cerebral microcirculation to hypertension.	Tarantini et al. (2016)
Adult CD-1 mice; MCAO	(1) Control, (2) AAV-GFP, (3) AAV-IGF-1	Post-stroke IGF-1 treatment can promote neural and vascular regeneration.	Zhu et al. (2009)
Igf1^f/f^; C57BL/6 male mice; Hypertension induced by Ang II	(1) Control, (2) Control + Ang II, (3) Control + SKF96365, (4) Igf1^f/f^ + TBG-iCre-AAV8, (5)Igf1^f/f^ + TBG-iCre-AAV8 + Ang II, (6) Igf1^f/f^ + TBG-iCre-AAV8 + SKF96365	IGF-1 deficiency induces functional maladaptation of cerebral arteries to hypertension, partly by the dysregulation of Transient receptor potential (TRP) channel.	Toth et al. (2014)

#### 3.1.1. Astrocytes

Astrocytes play important roles in neurotransmitter metabolism, synaptic transmission, nutritional support, blood–brain barrier function, neuroimmunity, and neurovascular remodeling (Stanimirovic and Friedman, 2012; Hort et al., 2019). During neuronal activity, astrocytes can signal directly to the brain’s perivascular endothelial cells through the endfeet, which in turn regulate cerebral blood flow (CBF) and ensure energy supply (Price et al., 2018).

Deficient IGF-1 signaling in astrocytes has been reported to decrease glutamate metabolism *in vitro* and *in vivo* by reducing the expression and availability of cell surface glutamate transporter proteins. Glutamate excitotoxicity is a major driver of neurodegeneration after ischemic stroke. Age-associated IGF-1 deficiency may also indirectly impair neurological function by altering the function of the supporting glial cells and vasculature (Prabhu et al., 2019; Hayes et al., 2021a). IGF-1 deficiency also impairs the production and release of vasomediator eicosanoids from astrocytes, alters endothelial nitric oxide (NO) production, and increases susceptibility to hypertension-induced microhemorrhage (Toth et al., 2015a; Tarantini et al., 2017; Fulop et al., 2019). Furthermore, IGF-1 affects glucose metabolism in astrocytes (Hernandez-Garzón et al., 2016) and, during the inflammatory response, degeneration of astrocytes and endfeet may occur due to matrix metalloproteinase (MMP) release degrading the dystrophin-dystroglycan complex that anchors the endfeet to the basement membrane of the vasculature (Weekman and Wilcock, 2016; Price et al., 2018; Hort et al., 2019). Moreover, IGF-1 has been shown to increase the number of astrocytes, connexins, and gap junctions (Åberg et al., 2003), and gene therapy has shown that IGF-1 increases astrocyte branching (Pardo et al., 2016).

#### 3.1.2. Oligodendrocytes

Oligodendrocytes play a key role in regulating neuronal excitability, maintaining and protecting normal neuronal function by wrapping around axons to form insulating myelin structures (Janowska et al., 2020).

Increasing evidence states an important role of IGF-1 in controlling oligodendrocyte function and the process of myelin production and regeneration (Mason et al., 2003; Zeger et al., 2007). In studies of ischemia–reperfusion injury, oligodendrocyte survival and myelin density were increased, and tissue swelling of white matter bundles was decreased in the sagittal parabasal gyrus within 90 min after IGF-1 administration, which may be related to apoptosis inhibition and increased oligodendrocyte proliferation (Guan et al., 2001; Cao et al., 2003). Although the protective effects of IGF-1 on oligodendrocytes have been demonstrated, the specific mechanisms involved remain to be investigated further.

#### 3.1.3. Endothelial cells

Endothelial cells form the lining of blood vessels and play an important role in the exchange of plasma and tissue fluid. In addition, they can control vasodilation and vasoconstriction by releasing vasoactive substances and balancing coagulation and anticoagulation. Endothelial dysfunction can result in several vascular pathologies, such as vascular stiffness, demyelination, and blood–brain barrier disruption (Maiuolo et al., 2018).

Evidence suggests that IGF-1 deficiency significantly impairs NO-dependent components of endothelial cells (Tarantini et al., 2021). Reduced endothelium-derived NO was observed in VCI and in a model of chronic cerebral hypoperfusion in hypertensive rats (Ren et al., 2018). In an animal model of IGF-1 deficiency, which usually exhibited increased reactive oxygen species (ROS) production and decreased NO bioavailability (Csiszar et al., 2008), IGF-1 treatment up-regulates endothelial nitric oxide synthase (eNOS) and increases NO bioavailability (Cittadini et al., 2009; Sonntag et al., 2013).

#### 3.1.4. Blood–brain barrier

Activation of the phosphatidylinositol 3-kinase-protein kinase B (PI3K-Akt) pathway has been shown to be activated by IGF-1 receptor stimulation, thereby attenuating the activation of glycogen synthase kinase-3β and mitogen-activated protein kinase kinase kinase 1 and increasing the expression of tight junction proteins (occludin and claudin-5), thus protecting the blood–brain barrier (Nowrangi et al., 2019). Animal models of deficient circulating IGF-1 usually exhibit impaired blood–brain barrier integrity (Hayes et al., 2021a), and in a stroke mouse model, IGF-1 infusion reduced blood–brain barrier permeability and decreased infarct volume caused by middle cerebral artery occlusion in middle-aged female rats (Bake et al., 2019). Recombinant IGF-1 injection improved blood–brain barrier permeability in a mouse model of intracerebral hemorrhage (Nowrangi et al., 2019).

### 3.2. Oxidative stress and inflammation

Many lines of evidence suggest that neuroinflammation is one of the primary pathophysiological mechanisms in cSVD, and microglia are the initiating factor in this process (Terasaki et al., 2014; Wardlaw et al., 2019). In the activated state, microglia have one of two cell phenotypes: M1 or M2. The M1 microglia have strong phagocytic capacity and secrete various pro-inflammatory factors, such as tumor necrosis factor-α (TNF-α), interleukin (IL)-6, inducible nitric oxide synthase (iNOS) and IL-1β, which enhance the inflammatory response and neurological injury, while the M2 microglia secrete anti-inflammatory mediators, such as IL-10 and transforming growth factor-β, which reduce inflammatory injury (Boche et al., 2013).

Numerous studies have shown that IGF-1 directly regulates microglial structure and function. Exogenous IGF-1 promotes M2 polarization of microglia, inhibits the M1 phenotype, alters the status of microglia phenotype activation, and reduces the production and release of microglia-associated TNF-α, IL-1β, iNOS, and ROS (Figure 2; Grinberg et al., 2013). Administration of IGF-1 after ischemic and/or hemorrhagic stroke attenuates microglial activation, inflammatory response, and ROS (Sohrabji and Williams, 2013; Serhan et al., 2019). A negative correlation between serum IL-6 and IGF-1 levels has been reported, and IGF/IGF binding protein-3 (IGFBP-3) administration to patients with severe burns induced anti-inflammatory effects and reduced IL-6 and TNF-α levels (Jeschke et al., 2000; Spies et al., 2002; Sukhanov et al., 2007; Higashi et al., 2012). IGF-1 deficiency has also been reported to exacerbate hypertension-induced oxidative stress, promote MMP activation, and lead to increased cerebral artery fragility, while antioxidant treatment attenuates age-associated hypertension-induced MMP activation (Tarantini et al., 2017). Moreover, IGF-1 can suppress the astrocyte response to inflammatory stimuli and protect neurons against neurotoxins (Labandeira-Garcia et al., 2017). The application of 1-methyl-4-phenyl-1,2,3,6-tetrahydropyridine in heterozygous IGF-1R mice performed more severe dopaminergic neuronal damage in the substantia nigra than it did in wild-type animals (Nadjar et al., 2009). Furthermore, exogenous IGF-I and IGF-I gene therapy inhibited the expression of toll-like receptor 4 and reduced the inflammatory response of astrocytes (Bellini et al., 2011).

### 3.3. Hemodynamics

#### 3.3.1. Microvascular rarefaction

Vascular density is closely associated with local blood perfusion. MVR reduces blood flow, which can lead to cerebral ischemic disease. Reduced availability of certain hormones and growth factors, including growth hormone, IGF-1, and vascular endothelial growth factor (VEGF), may contribute to MVR (Norling et al., 2020).

VEGF regulates microvascular density in the CNS, and its release is primarily induced by hypoxia-inducible factor-1α stimulated by IGF-1 (Figure 2; Lopez-Lopez et al., 2004; Sonntag et al., 2013). Animal and human studies suggest chronic VEGF inhibition leads to decreased endothelial cell survival, reduced blood flow, and vascular loss. Using IGF-1R-targeted antibodies to treat cancer patients can indirectly inhibit angiogenesis by suppressing VEGF production (Bid et al., 2012). Another study found that endocrine IGF-1 deficiency in IGF-1 knockout mice promoted hypertension-induced loss of hippocampal and neocortical microvessel density, which was reversed by IGF-1 infusion (Tarantini et al., 2016). Post-ischemic treatment with IGF-1 effectively promoted neural and vascular regeneration in the chronic stage of cerebral infarction. IGF-1 gene transfer significantly enhanced neovascularization and vascular density in the peri-infarct and injection needle tract area (Zhu et al., 2009).

#### 3.3.2. Regulation of CBF

Age-associated IGF-1 deficiency plays an important role in cerebral artery maladaptation to changes in the hemodynamic environment (Ungvari and Csiszar, 2012). Circulating IGF-1 levels decrease significantly with age and are associated with decreased basal CBF, and an age-related decrease in IGF-1 level may predict the magnitude of age-related decrease in neurovascular coupling response (Toth et al., 2022). Another study showed that hypertension in IGF-1-deficient mice is associated with impaired adaptive changes in myogenic constriction of cerebral arteries, mimicking the aging phenotype (Toth et al., 2014). Hypertension induction in IGF-1 knockout mice revealed impaired cerebrovascular autoregulation (Tarantini et al., 2017; Fulop et al., 2019). This functional maladaptation and increased microhemorrhage risk in hypertension may be related to the fact that IGF-1 deficiency disrupts the vascular remodeling process by impairing hypertension-induced adaptive media hypertrophy and dysregulating extracellular matrix remodeling, resulting in weakened cerebral arteries and increased circumferential stress (Tarantini et al., 2017; Norling et al., 2020).

## 4. Conclusion and outlook

In conclusion, serum IGF-1 levels might reflect the severity of cSVD and be associated with cognitive decline. Although IGF-1 may influence the formation and progression of cSVD in terms of NVU, CBF regulation, and neuroinflammation, evidence from clinical studies is lacking. The correlation between cSVD and IGF-1 is still being investigated, and it remains to be confirmed whether IGF-1 can be a predictor of cSVD by numerous basic and clinical studies. At the same time, these mechanisms are interrelated, and determining their specific order of occurrence is difficult. Therefore, the specific mechanisms by which IGF-1 affects cSVD remain to be elucidated.

A study found that stroke patients and high IGF1/IGFBP-3 ratios had better outcomes (Denti et al., 2004). Another study of the relationship between cognitive impairment and IGF-1 and IGFBP-3 found that plasma IGF-1 concentrations were not associated with dementia incidence, while doubling of plasma IGFBP-3 concentration reduced the risk ratio of dementia by 23% (Almeida et al., 2018). Therefore, future studies should probably not be limited to IGF-1 alone; the role of IGFBPs in regulating IGF-1 utilization may be a direction for further research.

## Author contributions

HD: investigation, data curation, and writing-original draft. JX, LH, and LZ: investigation and data curation. WG and FY: conceptualization, supervision, and writing-review and editing. All authors contributed to the article and approved the submitted version.

## Conflict of interest

The authors declare that the research was conducted in the absence of any commercial or financial relationships that could be construed as a potential conflict of interest.

## Publisher’s note

All claims expressed in this article are solely those of the authors and do not necessarily represent those of their affiliated organizations, or those of the publisher, the editors and the reviewers. Any product that may be evaluated in this article, or claim that may be made by its manufacturer, is not guaranteed or endorsed by the publisher.
